# Structural Validity of the Tonic Immobility Scale in a Population Exposed to Trauma: Evidence from Two Large Brazilian Samples

**DOI:** 10.1371/journal.pone.0094367

**Published:** 2014-04-18

**Authors:** Michael Reichenheim, Wanderson Souza, Evandro Silva Freire Coutinho, Ivan Figueira, Maria Inês Quintana, Marcelo Feijó de Mello, Rodrigo Affonseca Bressan, Jair de Jesus Mari, Sergio Baxter Andreoli

**Affiliations:** 1 Department of Epidemiology, Institute of Social Medicine (IMS), State University of Rio de Janeiro (UERJ), Rio de Janeiro, RJ, Brazil; 2 Department of Psychology, Institute of Education, Federal Rural University of Rio de Janeiro (UFRRJ), Seropédica, RJ, Brazil; 3 Department of Epidemiology, National School of Public Health (ENSP), FIOCRUZ, Rio de Janeiro, RJ, Brazil; 4 Institute of Psychiatry (IPUB), Federal University of Rio de Janeiro (UFRJ), Rio de Janeiro, RJ, Brazil; 5 Department of Psychiatry, Federal University of São Paulo (UNIFESP), São Paulo, SP, Brazil; Catholic University of Sacred Heart of Rome, Italy

## Abstract

**Background:**

Tonic Immobility is a temporary state of motor inhibition in situations involving extreme fear. The first scale developed for its assessment was the 10-item Tonic Immobility Scale (TIS). However, there are still few studies on its structural (dimensional) validity. The objective of this study was to reassess the factor structure of the TIS applied to representative samples exposed to general trauma of two Brazilian mega-cities.

**Methods:**

The sample comprised 3,223 participants reporting at least one traumatic experience. In São Paulo (n = 2,148), a Confirmatory Factor Analysis (CFA) first tested the originally proposed two-dimensional structure. This was followed by sequential Exploratory Structural Equation Models to identify the best fitting model, and subsequently tested in Rio de Janeiro (n = 1,075) via CFA. Alternative reduced versions were further explored using the aggregate sample. Model-based Item Response Theory (IRT) location parameters were also investigated.

**Results:**

An absence of factor-based convergent and discriminant validity rejected the original proposition. However, the one-dimensional structure still held several residual correlations. Further exploration indicated the sustainability of reduced versions with seven (alternative A) and six (alternative B) items. Both presented excellent fit and no relevant residual item correlation. According to the IRT location parameters, items in alternative B covered a wider range of the latent trait. The Loevinger's H scalability coefficients underscored this pattern.

**Conclusions:**

The original model did not hold. A one-factor solution was the most tenable in both large samples, but with significant item residual correlations, indicating that content redundancies persisted. Further reduced and simplified versions of the TIS proved promising. Although studies are yet to be carried out in other settings, it is the authors' impression that the restricted versions of the TIS are already apt for use in epidemiologic studies since the pros tend to outweigh the cons (as outlined in the Discussion section).

## Introduction

Despite extensively studied in the animal behavior literature, Tonic Immobility (TI) in humans is a recent area of research. TI is a temporary catatonic-like state marked by a reversible motor inhibition, muscle hypertonicity, analgesia and relative unresponsiveness to external stimuli. Some authors regard it an evolutionary adaptive component working as the terminal defensive reaction when other resources are unavailable [1], [2].

Usually called as a “playing dead” response in animals, TI is a consequence of a predatory attack in the wild when resistance is not successful [3], [4]. In laboratory studies, the induced state of immobility may persist from several seconds to hours after removal of restraint [1]. This response to threat seems to be evolutionarily beneficial, as it has been linked to higher survival rate to predatory attack in different species [5], [6]. Although most experiments in animal involve physical restraint, high fear circumstances that preclude escape may be sufficient for the induction of TI [2].

The majority of studies of TI in humans focuses on adult and childhood sexual assault as researchers previously hypothesized that reports of paralysis and inability to call out during assault experiences might be an expression of TI in humans [7]–[10]. However, there is a growing body of research reporting immobility symptoms in various contexts such as armed robbery/urban violence [11], trauma involving exposure to death/motor vehicle accidents [12], and even in air, nautical, and other disasters with non-interpersonal violence [13].

Along with other peritraumatic stress reactions, TI has been reported as a risk factor for Posttraumatic Stress Disorder – PTSD [10], [14], [15]. Fiszman et al. [11] showed that among victims of violence TI predicted the severity of posttraumatic stress symptoms, as well as a poor response to treatment. When comparing the effect of TI with other peritraumatic reactions, the literature presents contradictory results [12].

In spite of the growing interest in peritraumatic reactions, to the best of the authors' knowledge, there are only two measurement tools available to evaluate TI in humans. In 2009, Abrams et al. [16] proposed the Tonic Immobility Questionnaire TIQ designed to access human TI related to many traumatic events. Exploratory factor analysis suggested a three-factor solution, *viz.*, physical immobility, fear and dissociation.

The second and hitherto mostly used measurement tool is the Tonic Immobility Scale—Adult Form (hereafter referred to as TIS) presented by Forsyth et al. in 2000 [17], which is at the core of the present paper. Comprised of two parts, this self-report instrument was designed originally for evaluating the presence and severity of TI in female survivors of sexual assault. The first part assesses the dimensional aspects of the TI response while the second assesses victim and perpetrator behaviors that relate closely to sexual abuse experiences. The TI section consists of 11 items. Ten are rated on a seven point Likert-type ordinal scale [17]. According to the proponents, these are “10 face valid items that were derived from the animal literature”. To obtain the total score, item scores are added up (Table 1). The additional item evaluates earlier experiences concerning the 10 main component items, but are excluded from the scoring.

**Table 1:** Tonic Immobility Scale items used to compute the total score.

(1)	Rate the degree to which you froze or felt paralyzed during your most recent experience.
(2)	Rate the degree to which you were unable to move even though not restrained.
(3)	Rate the degree to which your body was trembling/shaking during the event.
(4)	Rate the degree to which you were unable to call out or scream during the event.
(5)	Rate the degree to which you felt numb or no pain during the event.
(6)	Rate the degree to which you felt cold during the event.
(7)	Rate the extent to which you felt feelings of fear/panic during the event.
(8)	Rate the extent to which you feared for your life or felt as though you were going to die.
(9)	Rate the extent to which you felt detached from yourself during the event.
(10)	Rate the extent to which you felt detached from what was going on around you during the event.

Fusé et al. [18] carried out an exploratory factor analysis of the TIS involving a sample of 88 victims of sexual abuse proposed two different latent factors labeled tonic immobility and anxiety. Accordingly, the fear factor would be composed of three items (fear/panic, trembling/shaking and feelings of detachment from surroundings), whereas the remaining seven items (froze/felt paralyzed, unable to move though not restrained, unable to call out or scream, felt numb/no pain, felt cold, feared for life and felt detached from self) would belong to the tonic immobility factor. An ensuing confirmatory factor analysis carried out on 191 victims of sexual assault purportedly corroborated this two-factor solution.

Although welcome as an opening to the scrutiny of the TIS, this psychometric history seems rather incipient and incongruous, especially when considering the time elapsed since its conception and given its continuous use over the years. For one, the evidence available so far arises from a domain too narrow (sexual abuse) to provide the TIS applicable to a broader population. Moreover, this evidence draws on relatively small samples. With an aim to redress these constraints and broaden the scope of use of the Tonic Immobility Scale, the goal of this study was to reassess its dimensional structure applied sequentially to large representative samples of two Brazilian mega-cities, São Paulo and Rio de Janeiro.

## Methods

### Ethics Statement

The Research Ethics Committee of the Federal University of São Paulo (Process No. 1369/04) approved the study in conformity with the principles embodied in the declaration of Helsinki. Participants were informed about research procedures and risks before signing an informed consent. Subjects who matched diagnostic criteria were offered referral to the outpatient clinic at the Federal University of São Paulo and Federal University of Rio de Janeiro.

### Sampling procedure and participants

The samples derive from two related surveys conducted from June/2007 to February/2008. The original design aimed to assess violence and mental health in the two largest Brazilian cities: São Paulo and Rio de Janeiro. A stratified (seven areas within the two cities ranked according to their homicide rates) multistage (census tracts, households, subjects) sampling procedure with unequal selection probabilities was carried out in both cities. See Andreoli et al. [19] for details.

Subjects from both representative samples were screened for history of trauma and stressful events. Exposure to a traumatic experience was assessed through a list of 32 events: 11 from the CIDI 2.1 [20] and 21 added by the authors [21], [22]. These additional events concerned episodes or situations effectively identifiable in the study domain. Some related to exposure to assaultive violence or other shocking events (e.g., being attacked with or without a weapon, death threats, having house broken in, experiencing parental and/or intimate partner violence), while others had to do with grief and suffering (e.g., sudden death or life-threatening illness of a close relative/person, car/motorcycle accident) [21], [22].

From the initial 3,744 participants, 3,239 (86%) reported at least one lifetime traumatic experience. The five most common ones were life-threatening illness of a close person (54%), sudden unexpected death of a close person (50%), seeing or touching a corpse (38%), being attacked with a weapon (38%) and witnessing someone being killed or injured (31%). A full account on all listed traumatic events is provided by Ribeiro et al. [22].

Those reporting at least one traumatic event gave further information about their peritraumatic symptoms and were screened for tonic immobility. Sixteen respondents provided ambiguous answers for at least one item of the scale and were, therefore, excluded from the analysis. The effective total sample size was thus of 3,223 participants (2,148 in São Paulo and 1,075 in Rio de Janeiro).

### Data Analysis

The dimensional evaluation initiated (step 1) by re-assessing the two-factor structure originally proposed by Fuse et al. (2007). A Confirmatory Factor Analysis (CFA) was first carried out on the São Paulo sample. This and all ensuing factor analyses employed the Mplus' robust weighted least squares mean and variance adjusted (WLSMV) estimator [23].Since the TIS comprises seven-level ordinal items, polychoric correlation matrices were suitably used as automatically generated in Mplus [24], [25]. Moreover, all analyses accounted for the complex sampling procedure involving stratification, clustering and unequal selection probabilities (sampling weights) [25], [26]. Goodness of fit was evaluated by three indices. The Root Mean Square Error of Approximation (RMSEA) is a model parsimony-adjusted fit index. Values close or below to .06 suggest an adequate fit [27]. The Comparative Fit Index (CFI) and the Tucker-Lewis Index (TLI) measure the improvement of fit by comparing the target model to a more restricted model. Both range from zero to one and values above 0.95 indicate adequate fit [27]. Factor-based discriminant analysis was also assessed by contrasting the square root of the Average Variance Extracted (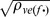) of each factor with the respective factor correlations [28], [29].

Anticipating a possible model misfit, step 2 consisted in re-evaluating the dimensional structure of the TIS through exploratory type analyses. First, eigenvalues were examined through an Exploratory Common Factor Analysis (EFA) [30]. Depending on the findings, one or several sequential Exploratory Structural Equation Models (ESEM) would be fitted [31]. These models consist of exploratory models estimated within a CFA framework (a.k.a. E/CFA) and offer the advantage over the traditional EFA models in that they also allow for assessing other relevant features as, for instance, potential item residual correlations (which may arise from item content redundancies). These were explored through Modification Indices (MI), which reflect how much the overall model chi-square would decrease if a constrained parameter were freely estimated. The E/CFAs used Geomin oblique rotations.

The ‘best’ re-specified dimensional structure identified in the São Paulo sample was then tested on the independent sample collected in Rio de Janeiro, again using a CFA (step 3). To complete the process, we tentatively explored the tenability of reduced versions of the instrument, given several item residual correlations could be uncovered (as fully outlined in the Results section). Beyond the features explores in previous models, we also investigated the Item Response Theory (IRT) model-based location parameters of the restricted versions [29], [32]. These *b_i_* parameters are useful to indicate how well items map the alleged latent trait in terms of its increasing intensity or severity [29], [33]. Provided both the assumptions of single dimensionality and conditional (local) independence could be ascertained, the parameters were calculated directly from the CFA loadings and thresholds through 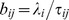, where subscript *i* refers to items and *j* indicates related cut-off points [29]. We also examined the appropriateness of raw scores as pragmatic proxy measure for ranking respondents along the overall latent trait by assessing their correlations with the model-based factor scores [34]. The former scores were obtained by the sums of item raw scores (*X*_+_) whereas the latter were estimated from the respective CFA models via maximum a posteriori method as implemented in Mplus [25]. Finally, scalability was assessed using Loevinger's H [34] using a special Stata routine [35], [36]. As suggested by Mokken, values >0.3 indicate acceptable levels [apud 34].

## Results

Table 2 provides the samples' age and sex distribution. More women reported a positive history of trauma. The sample in Rio de Janeiro was slightly older than in São Paulo.

**Table 2:** Sex distribution and age mean by city and aggregate.

	Women		Men	
**Aggregate**				
Percentage	56.4	(54.8–58.1)	43.6	(41.9–45.2)
Mean age	41.1	(38.5–42.0)	39.5	(41.9–45.2)
**São Paulo**				
Percentage	56.8	(54.7–58.8)	43.2	(41.2–45.3)
Mean age	40.0	(38.9–41.1)	39.0	(37.7–40.4)
**Rio de Janeiro**				
Percentage	55.7	(53.1–58.3)	44.3	(41.7–46.9)
Mean age	43.6	(42.2–45.0)	40.8	(39.1–42.4)

Note: estimates and 95% C.I. in brackets account for complex sampling procedure (see text for details).

The originally proposed two-factor CFA solution showed a poor fit. As shown in Table 3(A), the RMSEA was above acceptable levels, especially concerning the upper bound. Additionally, this model presented a factor correlation of 0.980, far higher than the square roots of the average variance extracted of each factor. The MIs also suggested several residual correlations to explore.

**Table 3:** Sequence of models concerning the Tonic Immobility Scale (TIS).

		Model A (original)^1^		Model B^1^	Model C^1^	Model D^2^
		2-factor CFA		1-factor – ESEM*	1-factor ESEM**	1-factor CFA
		Factor 1	Factor 2	Factor 1	Factor 1	Factor 1
i1.	*Frozen/paralyzed*	.837	—	.826	.791	.847
i2.	*Unable to move*	.833	—	.836	.793	.872
i3.	*Shaking*	—	.764	.757	.750	.791
i4.	*Unable to vocalize*	.786	—	.773	.793	.814
i5.	*Numb/no pain*	.775	—	.777	.794	.751
i6.	*Felt cold*	.741	—	.742	.758	.781
i7.	*Fear/panic*	—	.741	.733	.708	.729
i8.	*Felt like dying*	.664	—	.664	.645	.604
i9.	*Detached from self*	.793	—	.792	.741	.742
i10.	*Detached from environment*		.697	.690	.626	.658
	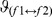	.980 (.960–1.00)		—	—	—
	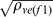	.698 (.681–.714)		—	—	—
	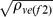	.766 (.757–.775)		—	—	—
						
	*i1*↔*i4*	−.202		—	—	—
	*i1*↔*i2*	—		—	.422	.172
	*i3*↔*i7*	—		—	.264	.363
	*I7*↔*i8*	—		—	.318	.349
	*I9*↔*i10*	—		—	.528	.436
	RMSEA^a^	.073 (.066; .079)		.071 (.065; .077)	.027 (.019; .034)	.033 (.022; .044)
	CFI^b^	.972		.972	.996	.993
	TLI^c^	.962		.964	.995	.990

1 São Paulo sample (n = 2148).

2 Rio de Janeiro sample (n = 1075). All estimates account for the complex sampling procedure (see text for details).

* Suggested expected parameter changes for residual correlations: i1↔i2 = .609, i2↔i7 = −.274, i3↔i7 = .313, i7↔i8 = .351 and i9↔i10 = .680.

** ESEM (E/CFA) with item residual error correlation freely estimated.

a RMSEA = Root Mean Square Error of Approximation; In brackets: 90% confidence intervals.

b CFI = Comparative Fit Index.

c TLI = Tuker-Lewis Index.

The EFA fitted in the following step revealed only one eigenvalue above one (eig_(f1)_ = 6.072, eig_(f2)_ = .824 or eig_(f3)_ = .662, …). The two-factor E/CFA showed eight items loading on a main factor while only items 9 and 10 loading on a second factor. Although no relevant cross-loadings were detected, this model still did not fit adequately (e.g., RMSEA = .058; 90% CI = .065) while the high factor correlation persisted (F1↔F2 = .695). In the three-factor E/CFA, model fit improved (RMSEA = .038), factor correlations somehow decreased (F1↔F2 = .752; F1↔F3 = .657 and F1↔F3 = .710), but loadings attenuated sharply, and a few cross loadings emerged.

One option was thus to pursue the exploration of one-dimensional structures. The strict one-factor E/CFA (Model B in Table 3) showed a poor model fit (RMSEA = .071) and the MIs suggested five residual correlations (i1↔i2, i2↔i7,i3↔i7, i7↔i8 and i9↔i10). As shown in Model C of Table 3, four of those hold up once freely estimated. Model fit improved substantially in all indices (RMSEA = .027, CFI = .996 and TLI = .995), reinforcing possible item content redundancies needing further corroboration on new data set as followed.

Using the Rio de Janeiro sample, a CFA model was then fitted to the one-dimensional solution suggested in this last ESEM fitted on the São Paulo sample (Model D in Table 3).Factor loadings were moderate to high and the same four residual correlations persisted. Model fit was also satisfactory.

Given the recurrence of the residual correlations in the data from Rio de Janeiro, we further explored other models with reduced item sets. Using the São Paulo and Rio aggregate data, two alternatives were sought. In both, items i2 and i9 were retained since they had the highest loadings in the respective pairs (i1↔i2 and i9↔i10). Item 7 was removed in Alternative A since its error correlated with both i3 and i8. For the same reason, item 7 was kept in Alternative B, but the other two —i3 and i8— were dropped in turn. Regardless, these reduced versions presented excellent fit and no relevant residual item correlation as conveyed in Table 4.

**Table 4:** Alternative restricted models applied to the Rio de Janeiro sample excluding Tonic Immobility Scale's redundant items: Confirmatory Factor Analysis loadings, and items' and scale assessment of scalability via Loevinger's H coefficient.

		CFA loadings		Loevinger's H coefficient	
		Alternative A	Alternative B	Alternative A	Alternative B
i1.	*Frozen/paralyzed*	—	—	—	—
i2.	*Unable to move*	.823	.819	.482	.504
i3.	*Shaking*	.755	—	.485	—
i4.	*Unable to vocalize*	.802	.800	.461	.489
i5.	*Numb/no pain*	.780	.790	.454	.483
i6.	*Felt cold*	.772	.760	.446	.464
i7.	*Fear/panic*	—	.703	—	.532
i8.	*Felt like dying*	.641	—	.382	—
i9.	*Detached from self*	.742	.758	.449	.482
i10.	*Detached from environment*	—	—	—	—
	Full scale	(n.a.)	(n.a.)	.452	.493
	RMSEA^a^	.033 (.025; .041)	.029 (.018; .039)		
	CFI^b^	.996	.997		
	TLI^c^	.993	.996		

Note: items 1 (“*Froze or paralyzed*”) and 10 (“*Detachment from surroundings*”) removed from both alternative scales.

**Figure 1:**
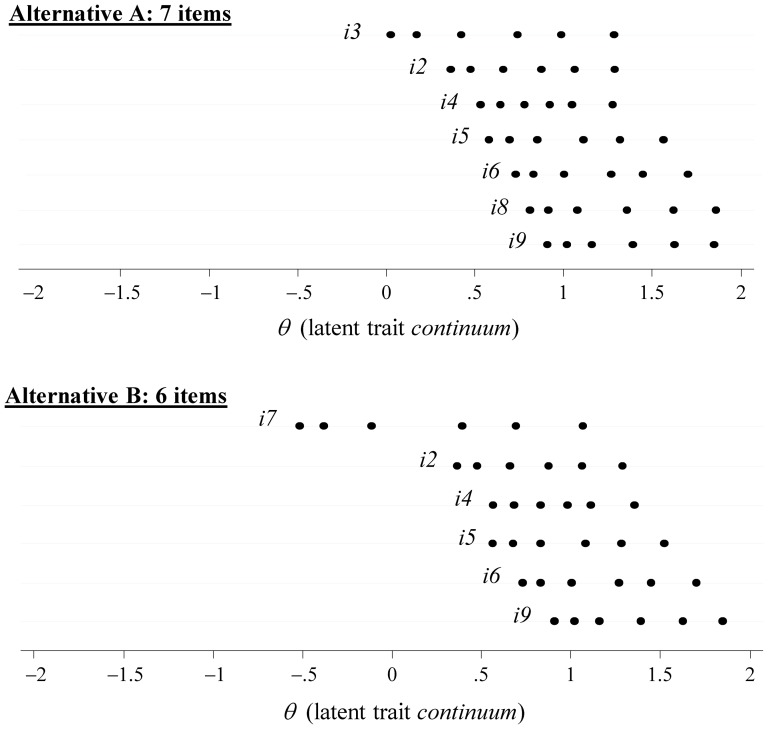
Item thresholds (6 *per* item) dispersion along the *θ* latent trait *continuum* (factor score) pertaining to the seven-level items (6 thresholds) of the reduced TIS version. Aggregate São Paulo and Rio de Janeiro sample.

However, as conveyed in Figure 1, there are differences regarding how the items of the reduced versions map the latent trait continuum. Represented by the IRT location parameters, both within and between items, the *b_ij_* rise along the *θ* latent trait spectrum in both models, but that items cover a wider range in Alternative B. This is mainly due to the retention of i7, which clearly stretches further into an area of ‘milder intensity’ (lower *θ* values). The Loevinger's H scalability coefficients underscore this pattern. Returning to Table 4, although both coefficients are above the cutoff point suggested by Mokken (0.3), H_B_ is 8.3% higher than H_A_. The relative strength of Alternative B may also be perceived when comparing all item-specific H coefficients. An additional feature concerns the high correlations between the raw scores and the extracted factor scores: *ρ_(A)_* = .965 (95% CI: .963–.967) and *ρ_(B)_* = .970 (95% CI: .968–.972).

## Discussion

As conveyed in the introduction, although the TIS has been used in various settings, its dimensional structure has only been evaluated in the narrow domain of sexually abused women and by studies using rather small sample sizes. This study, in contrast, was carried out in a large representative population sample subjected to a variety of traumatic experiences. This may perhaps explain the differences between findings. For one, the two-factor structure did not hold since the factor based discriminant validity was far from acceptable when attempting to fit the original model proposed by Fusé et al. [18].

The one-factor solution was the most tenable in both large samples, although several observed residual correlations suggested item content redundancies. These are theoretically meaningful when examining the connotative contents of each item pair. The first set concerns items 1 and 2, both expressing the idea of immobility itself. One could argue that *feeling paralyzed* or *frozen* mean something more than the simple incapacity to move. Nevertheless, we found no support to this hypothesis suggesting that either the two expressions have the same connotation or the individuals interpreted them as synonyms.

The second set involves two pairs and three items, namely, i3 (*trembling/shaking*), i7 (*fear/panic*) and i8 (*feared for life*). The content overlap of i3 and i7 is hardly surprising since trembling and shaking are one of the most commonly recognized physical expressions of fear and panic. Expected, too, is the second overlap involving i7 and i8 given both items use the term *fear* in their wording structure. Possibly, what respondents make of the items' joint content converges to the idea of “trembling with fear”, which, incidentally, is a very common saying in the study setting.

As in the i7–i8 pair, shared wording may also explain the content intersection of i9 (detached from self) and i10 (detached from environment). In common, both behold the feeling of detachment, which is more related to the concept of dissociation than immobility. In passing, more research may shed some light on this last point, especially regarding the development of an instrument specifically tailored to assess immobility, perhaps containing more items on fear and related events. More accurate and focused measurement tools could promote a better evaluation and understanding of tonic immobility reaction and, by extension, its relationship with other peritraumatic reactions.

The meeting of various residual correlations led to the initiative to seek some simplification. Although both tentative models turned out appropriate, alternative model B looks better; not only does it holds fewer items and is thus more parsimonious, but also it enhances content coverage and scalability. Regardless, restricting items in the situation at hand may be auspicious for two reasons. For one, it would increase efficiency by lessening the duration of the interview, which is an almost ubiquitous requirement in large studies involving multi-faceted questionnaires. Secondly, avoiding correlated residuals clarifies if the scale is to be eventually used in its raw score format (as often happens in applied research contexts). Items holding redundancies may lead to metric ‘overweighting’ since their shared (overlapped) contents are not accounted for in the total *X*_+_ raw score.

The differences between the one-factor solution and the two-factor solution proposed by Fusé (2007) might be a result of methodological issues (sample size), domain issues (sexually abused women vs community settings) or even cultural particularities. Despite these differences, it is auspicious that the TIS showed suitable for also for general populations exposed to a large variety of traumas (see Ribeiro et al [22]).

The results of this study must be seen in the light of their strengths and weakness. On the positive side stand the large samples arising from two large cities holding similar yet comprehensive domains, which enhances precision and generalizability. Secondly, all analysis took into account the complex sampling process. Thirdly, the study involved testing the instrument in two separate populations, the high consistency of findings between the ‘exploration’ (São Paulo) and ‘confirmation’ (Rio de Janeiro) samples being of most interest. Admittedly, though, the present finding are confined to a particular social milieu. Cultural determination should not be overlooked; there is always the possibility that some findings fail to replicate, which is a reason to put the current models to new testing. Another issue requiring attention is that the TIS was applied outside its original development context. Based on predator-prey relationships found in the animal world as reflecting particular trauma related to sexual assault, the TIS was tested here in a wider population subjected to a variety of traumas. Perhaps, it would be desirable also to adjust the instrument so that the reactions become tuned in with this diversity. It would thus be desirable to delve into adjusting the instrument further so that the reactions become fine-tuned with this diversity.

Since this seems a long run prospect, particular studies should be carried out in other community populations and domains in order to evaluate critically the reduced format suggested in this paper. For the time being, though, it is the authors' impression that a reduced version of the TIS is already a viable option for use in epidemiologic studies since that the pros tend to outweigh the cons.
